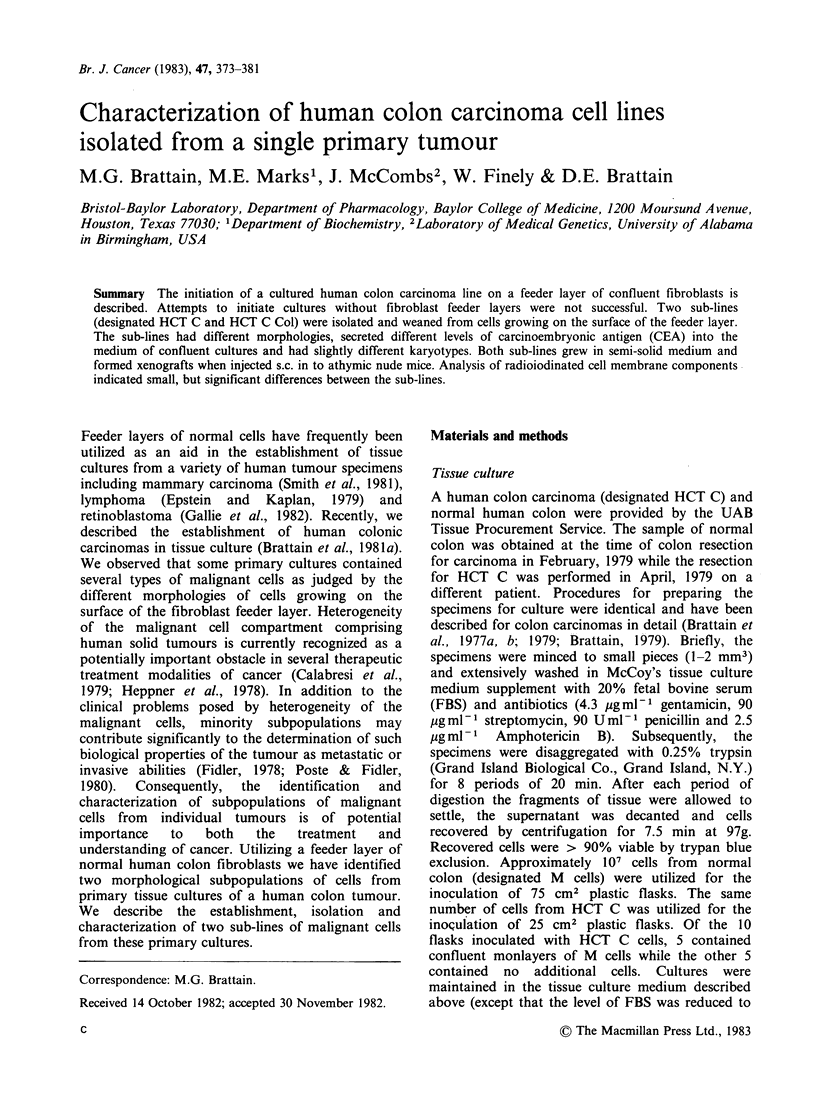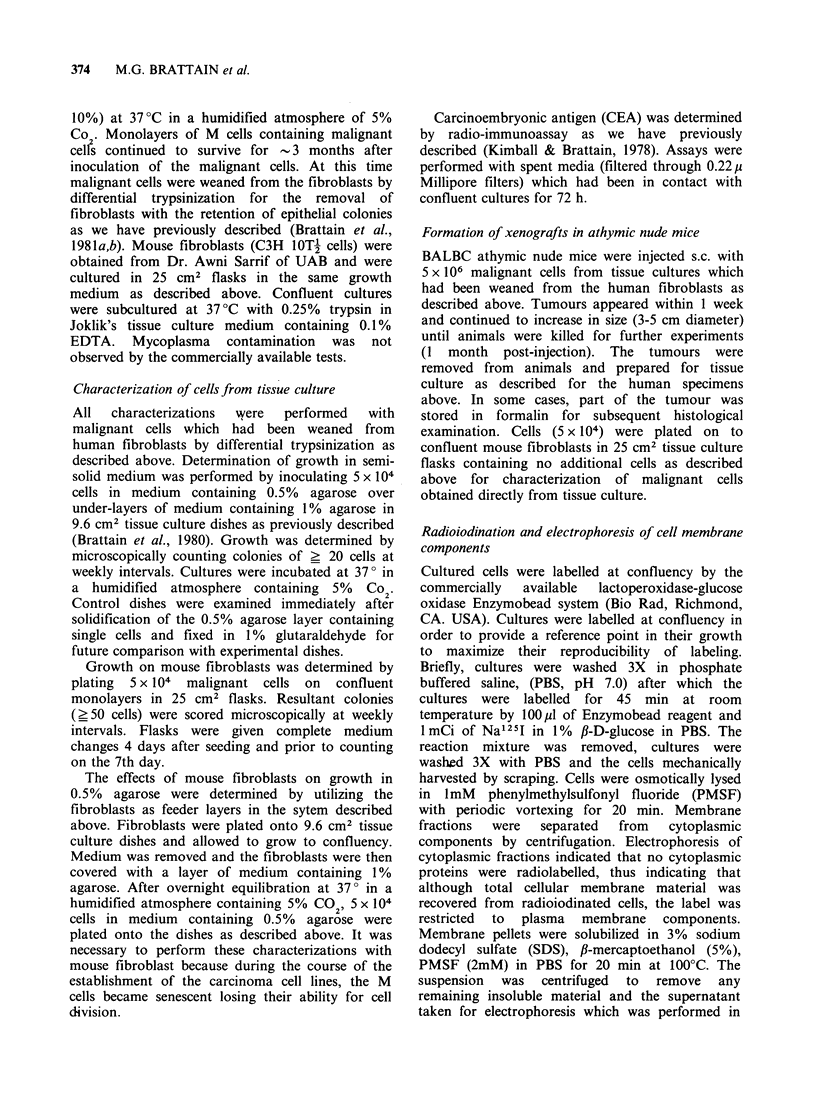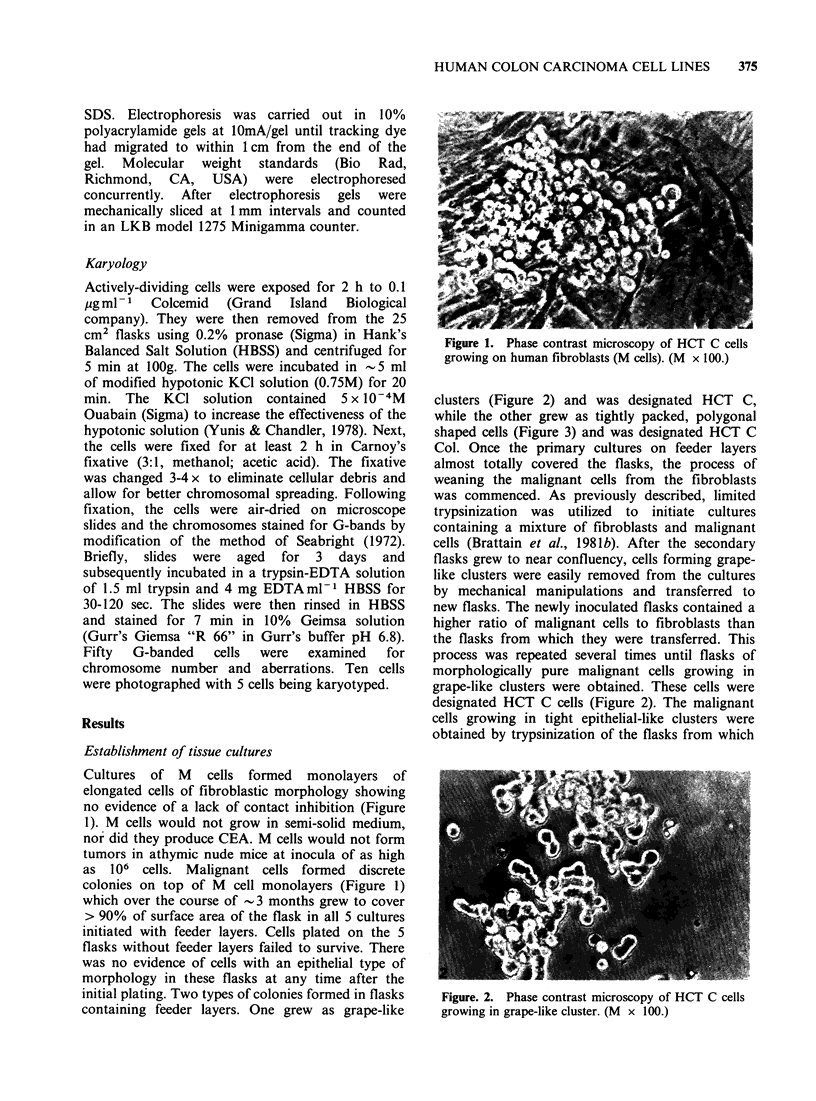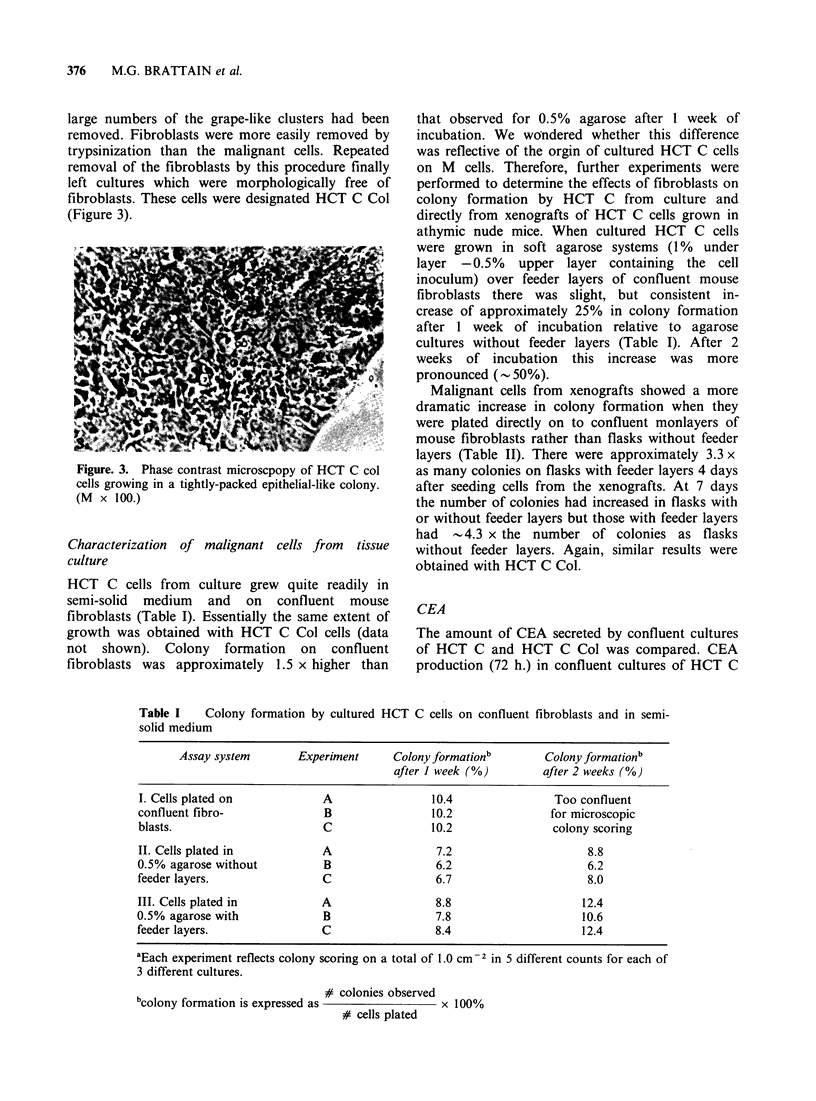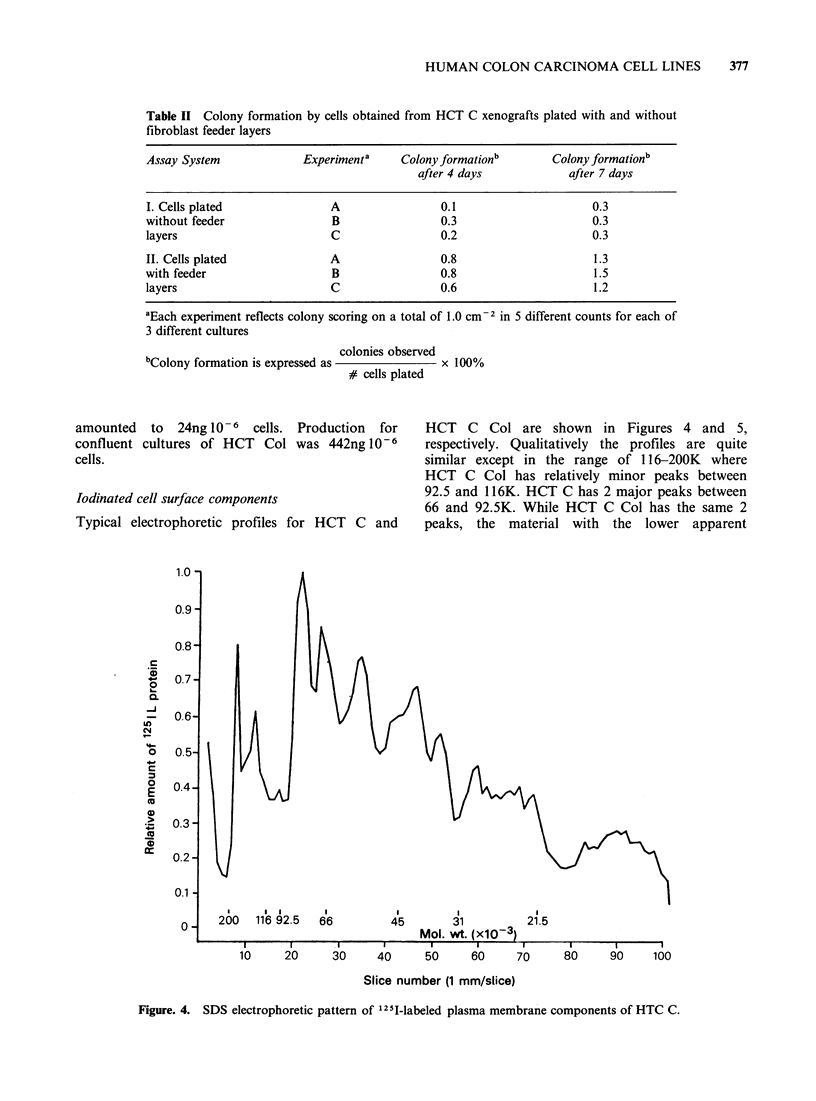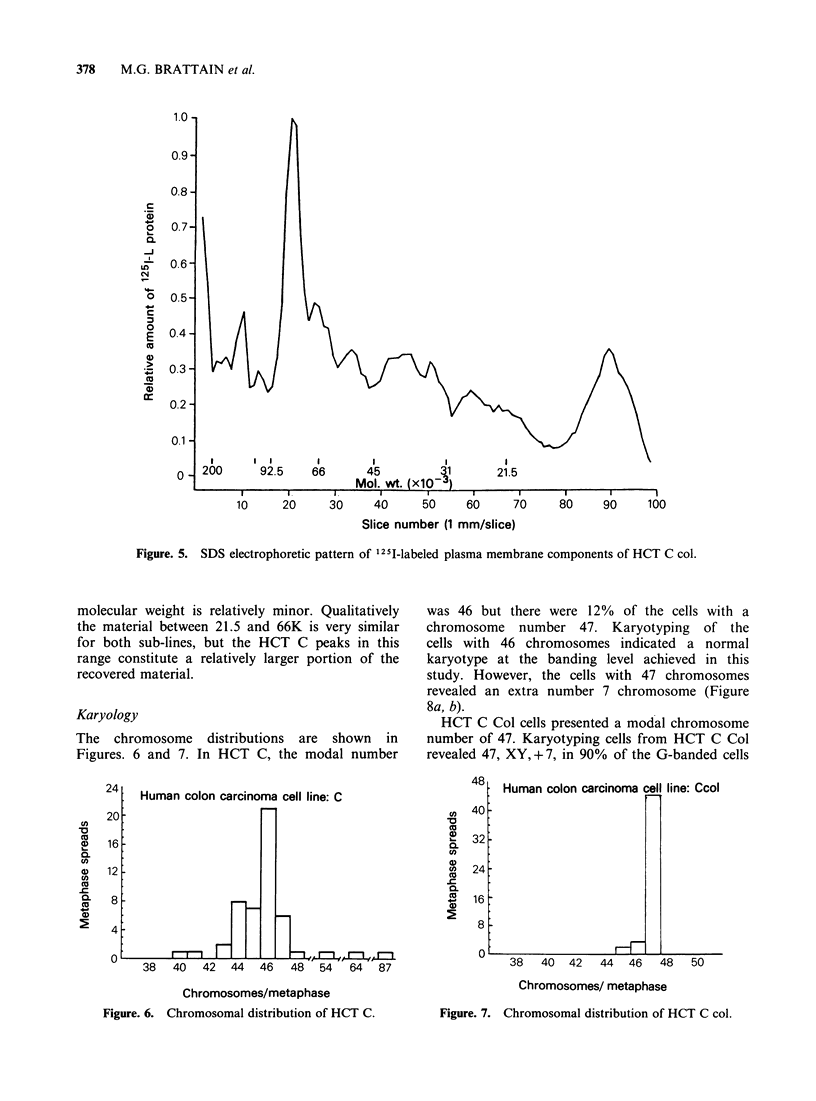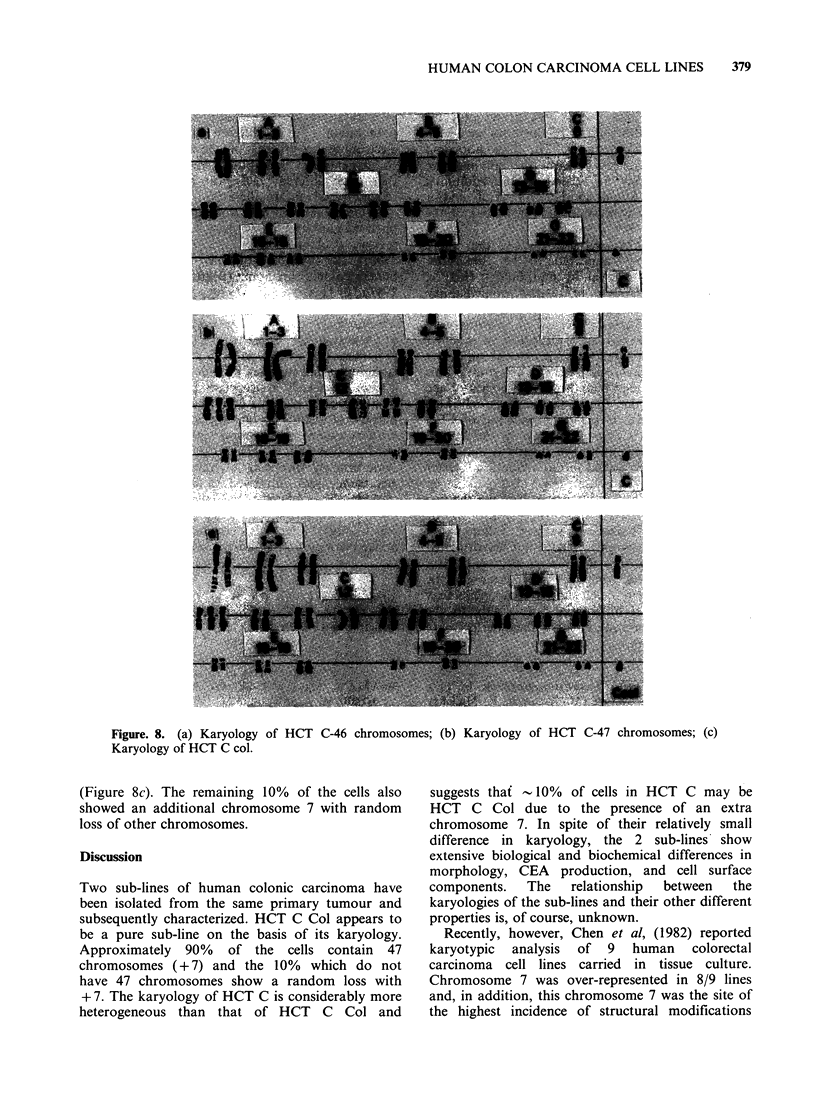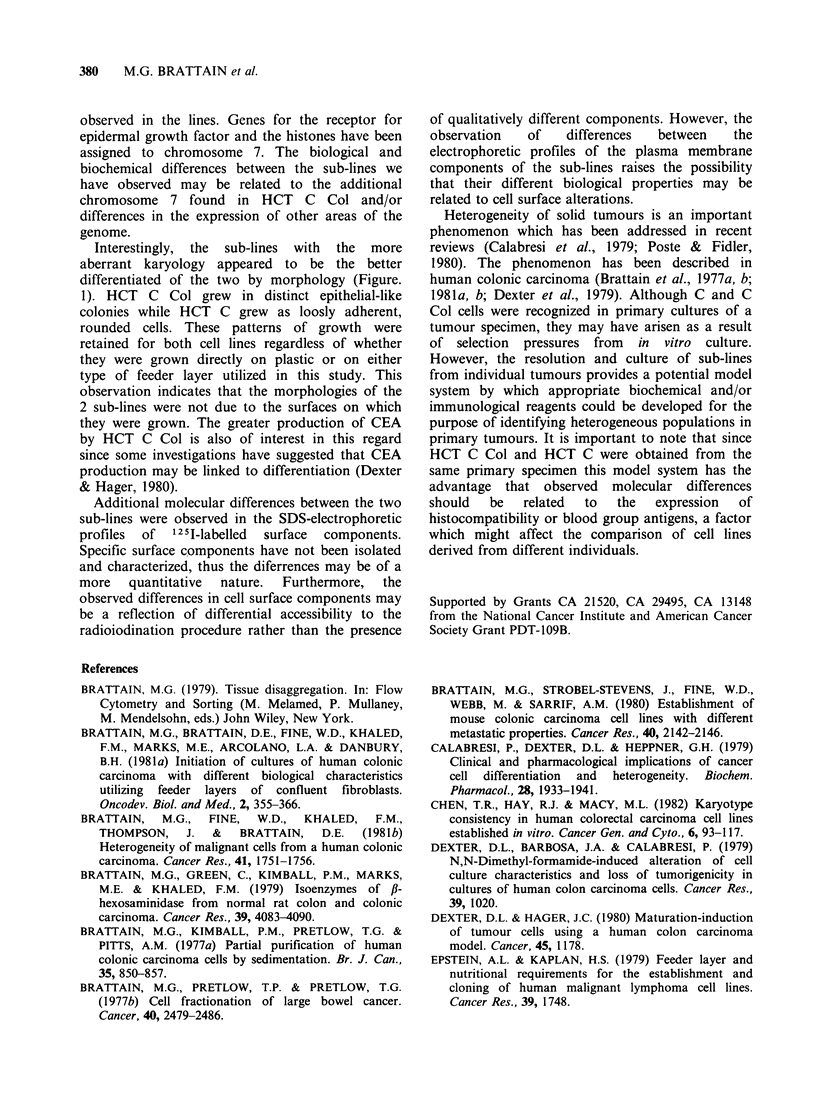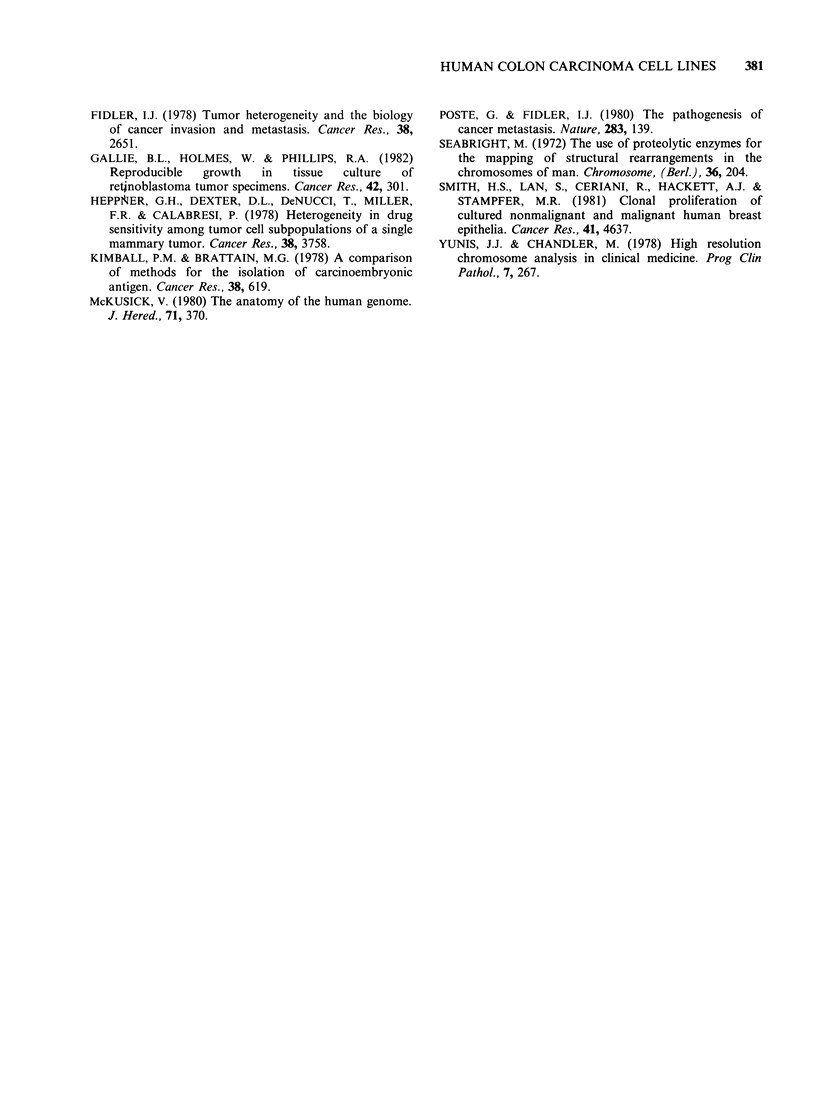# Characterization of human colon carcinoma cell lines isolated from a single primary tumour.

**DOI:** 10.1038/bjc.1983.56

**Published:** 1983-03

**Authors:** M. G. Brattain, M. E. Marks, J. McCombs, W. Finely, D. E. Brattain

## Abstract

**Images:**


*Br. J. Cancer* (1983), **47**, 373–381

### Characterization of human colon carcinoma cell lines isolated from a single primary tumour

M.G. Brattain, M.E. Marks[1], J. McCombs[2], W. Finely & D.E. Brattain

*Bristol-Baylor Laboratory, Department of Pharmacology, Baylor College of Medicine, 1200 Moursund Avenue, Houston, Texas 77030; [1]Department of Biochemistry, [2]Laboratory of Medical Genetics, University of Alabama in Birmingham, USA*

**Summary** The initiation of a cultured human colon carcinoma line on a feeder layer of confluent fibroblasts is described. Attempts to initiate cultures without fibroblast feeder layers were not successful. Two sub-lines (designated HCT C and HCT C Col) were isolated and weaned from cells growing on the surface of the feeder layer. The sub-lines had different morphologies, secreted different levels of carcinoembryonic antigen (CEA) into the medium of confluent cultures and had slightly different karyotypes. Both sub-lines grew in semi-solid medium and formed xenografts when injected s.c. in to athymic nude mice. Analysis of radioiodinated cell membrane components indicated small, but significant differences between the sub-lines.

Feeder layers of normal cells have frequently been utilized as an aid in the establishment of tissue cultures from a variety of human tumour specimens including mammary carcinoma (Smith *et al.*, 1981), lymphoma (Epstein and Kaplan, 1979) and retinoblastoma (Gallie *et al.*, 1982). Recently, we described the establishment of human colonic carcinomas in tissue culture (Brattain *et al.*, 1981*a*). We observed that some primary cultures contained several types of malignant cells as judged by the different morphologies of cells growing on the surface of the fibroblast feeder layer. Heterogeneity of the malignant cell compartment comprising human solid tumours is currently recognized as a potentially important obstacle in several therapeutic treatment modalities of cancer (Calabresi *et al.*, 1979; Heppner *et al.*, 1978). In addition to the clinical problems posed by heterogeneity of the malignant cells, minority subpopulations may contribute significantly to the determination of such biological properties of the tumour as metastatic or invasive abilities (Fidler, 1978; Poste & Fidler, 1980). Consequently, the identification and characterization of subpopulations of malignant cells from individual tumours is of potential importance to both the treatment and understanding of cancer. Utilizing a feeder layer of normal human colon fibroblasts we have identified two morphological subpopulations of cells from primary tissue cultures of a human colon tumour. We describe the establishment, isolation and characterization of two sub-lines of malignant cells from these primary cultures.

Correspondence: M.G. Brattain.

Received 14 October 1982; accepted 30 November 1982.

### Materials and methods

### *Tissue culture*

A human colon carcinoma (designated HCT C) and normal human colon were provided by the UAB Tissue Procurement Service. The sample of normal colon was obtained at the time of colon resection for carcinoma in February, 1979 while the resection for HCT C was performed in April, 1979 on a different patient. Procedures for preparing the specimens for culture were identical and have been described for colon carcinomas in detail (Brattain *et al.*, 1977*a*, *b*; 1979; Brattain, 1979). Briefly, the specimens were minced to small pieces (1–2 $mm^3$) and extensively washed in McCoy's tissue culture medium supplement with 20% fetal bovine serum (FBS) and antibiotics (4.3 $\mu g\,ml^{-1}$ gentamicin, 90 $\mu g\,ml^{-1}$ streptomycin, 90 $U\,ml^{-1}$ penicillin and 2.5 $\mu g\,ml^{-1}$ Amphotericin B). Subsequently, the specimens were disaggregated with 0.25% trypsin (Grand Island Biological Co., Grand Island, N.Y.) for 8 periods of 20 min. After each period of digestion the fragments of tissue were allowed to settle, the supernatant was decanted and cells recovered by centrifugation for 7.5 min at 97g. Recovered cells were > 90% viable by trypan blue exclusion. Approximately $10^7$ cells from normal colon (designated M cells) were utilized for the inoculation of 75 $cm^2$ plastic flasks. The same number of cells from HCT C was utilized for the inoculation of 25 $cm^2$ plastic flasks. Of the 10 flasks inoculated with HCT C cells, 5 contained confluent monlayers of M cells while the other 5 contained no additional cells. Cultures were maintained in the tissue culture medium described above (except that the level of FBS was reduced to

© The Macmillan Press Ltd., 1983

10%) at 37 °C in a humidified atmosphere of 5% $Co_2$. Monolayers of M cells containing malignant cells continued to survive for ~3 months after inoculation of the malignant cells. At this time malignant cells were weaned from the fibroblasts by differential trypsinization for the removal of fibroblasts with the retention of epithelial colonies as we have previously described (Brattain *et al.*, 1981*a,b*). Mouse fibroblasts (C3H 10T$\frac{1}{2}$ cells) were obtained from Dr. Awni Sarrif of UAB and were cultured in 25 $cm^2$ flasks in the same growth medium as described above. Confluent cultures were subcultured at 37 °C with 0.25% trypsin in Joklik's tissue culture medium containing 0.1% EDTA. Mycoplasma contamination was not observed by the commercially available tests.

*Characterization of cells from tissue culture*

All characterizations were performed with malignant cells which had been weaned from human fibroblasts by differential trypsinization as described above. Determination of growth in semi-solid medium was performed by inoculating $5 \times 10^4$ cells in medium containing 0.5% agarose over under-layers of medium containing 1% agarose in 9.6 $cm^2$ tissue culture dishes as previously described (Brattain *et al.*, 1980). Growth was determined by microscopically counting colonies of $\geqq$ 20 cells at weekly intervals. Cultures were incubated at 37° in a humidified atmosphere containing 5% $Co_2$. Control dishes were examined immediately after solidification of the 0.5% agarose layer containing single cells and fixed in 1% glutaraldehyde for future comparison with experimental dishes.

Growth on mouse fibroblasts was determined by plating $5 \times 10^4$ malignant cells on confluent monolayers in 25 $cm^2$ flasks. Resultant colonies ($\geqq$50 cells) were scored microscopically at weekly intervals. Flasks were given complete medium changes 4 days after seeding and prior to counting on the 7th day.

The effects of mouse fibroblasts on growth in 0.5% agarose were determined by utilizing the fibroblasts as feeder layers in the sytem described above. Fibroblasts were plated onto 9.6 $cm^2$ tissue culture dishes and allowed to grow to confluency. Medium was removed and the fibroblasts were then covered with a layer of medium containing 1% agarose. After overnight equilibration at 37° in a humidified atmosphere containing 5% $CO_2$, $5 \times 10^4$ cells in medium containing 0.5% agarose were plated onto the dishes as described above. It was necessary to perform these characterizations with mouse fibroblast because during the course of the establishment of the carcinoma cell lines, the M cells became senescent losing their ability for cell division.

Carcinoembryonic antigen (CEA) was determined by radio-immunoassay as we have previously described (Kimball & Brattain, 1978). Assays were performed with spent media (filtered through 0.22 $\mu$ Millipore filters) which had been in contact with confluent cultures for 72 h.

*Formation of xenografts in athymic nude mice*

BALBC athymic nude mice were injected s.c. with $5 \times 10^6$ malignant cells from tissue cultures which had been weaned from the human fibroblasts as described above. Tumours appeared within 1 week and continued to increase in size (3-5 cm diameter) until animals were killed for further experiments (1 month post-injection). The tumours were removed from animals and prepared for tissue culture as described for the human specimens above. In some cases, part of the tumour was stored in formalin for subsequent histological examination. Cells ($5 \times 10^4$) were plated on to confluent mouse fibroblasts in 25 $cm^2$ tissue culture flasks containing no additional cells as described above for characterization of malignant cells obtained directly from tissue culture.

*Radioiodination and electrophoresis of cell membrane components*

Cultured cells were labelled at confluency by the commercially available lactoperoxidase-glucose oxidase Enzymobead system (Bio Rad, Richmond, CA. USA). Cultures were labelled at confluency in order to provide a reference point in their growth to maximize their reproducibility of labeling. Briefly, cultures were washed 3X in phosphate buffered saline, (PBS, pH 7.0) after which the cultures were labelled for 45 min at room temperature by 100 $\mu$l of Enzymobead reagent and 1 mCi of $Na^{125}I$ in 1% $\beta$-D-glucose in PBS. The reaction mixture was removed, cultures were washed 3X with PBS and the cells mechanically harvested by scraping. Cells were osmotically lysed in 1mM phenylmethylsulfonyl fluoride (PMSF) with periodic vortexing for 20 min. Membrane fractions were separated from cytoplasmic components by centrifugation. Electrophoresis of cytoplasmic fractions indicated that no cytoplasmic proteins were radiolabelled, thus indicating that although total cellular membrane material was recovered from radioiodinated cells, the label was restricted to plasma membrane components. Membrane pellets were solubilized in 3% sodium dodecyl sulfate (SDS), $\beta$-mercaptoethanol (5%), PMSF (2mM) in PBS for 20 min at 100°C. The suspension was centrifuged to remove any remaining insoluble material and the supernatant taken for electrophoresis which was performed in

SDS. Electrophoresis was carried out in 10% polyacrylamide gels at 10mA/gel until tracking dye had migrated to within 1 cm from the end of the gel. Molecular weight standards (Bio Rad, Richmond, CA, USA) were electrophoresed concurrently. After electrophoresis gels were mechanically sliced at 1 mm intervals and counted in an LKB model 1275 Minigamma counter.

*Karyology*

Actively-dividing cells were exposed for 2 h to 0.1 $\mu g\,ml^{-1}$ Colcemid (Grand Island Biological company). They were then removed from the 25 $cm^2$ flasks using 0.2% pronase (Sigma) in Hank's Balanced Salt Solution (HBSS) and centrifuged for 5 min at 100g. The cells were incubated in $\sim 5$ ml of modified hypotonic KCl solution (0.75M) for 20 min. The KCl solution contained $5 \times 10^{-4}$M Ouabain (Sigma) to increase the effectiveness of the hypotonic solution (Yunis & Chandler, 1978). Next, the cells were fixed for at least 2 h in Carnoy's fixative (3:1, methanol; acetic acid). The fixative was changed 3-4 $\times$ to eliminate cellular debris and allow for better chromosomal spreading. Following fixation, the cells were air-dried on microscope slides and the chromosomes stained for G-bands by modification of the method of Seabright (1972). Briefly, slides were aged for 3 days and subsequently incubated in a trypsin-EDTA solution of 1.5 ml trypsin and 4 mg EDTA $ml^{-1}$ HBSS for 30-120 sec. The slides were then rinsed in HBSS and stained for 7 min in 10% Geimsa solution (Gurr's Giemsa "R 66" in Gurr's buffer pH 6.8). Fifty G-banded cells were examined for chromosome number and aberrations. Ten cells were photographed with 5 cells being karyotyped.

### Results

*Establishment of tissue cultures*

Cultures of M cells formed monolayers of elongated cells of fibroblastic morphology showing no evidence of a lack of contact inhibition (Figure 1). M cells would not grow in semi-solid medium, nor did they produce CEA. M cells would not form tumors in athymic nude mice at inocula of as high as $10^6$ cells. Malignant cells formed discrete colonies on top of M cell monolayers (Figure 1) which over the course of $\sim 3$ months grew to cover $> 90\%$ of surface area of the flask in all 5 cultures initiated with feeder layers. Cells plated on the 5 flasks without feeder layers failed to survive. There was no evidence of cells with an epithelial type of morphology in these flasks at any time after the initial plating. Two types of colonies formed in flasks containing feeder layers. One grew as grape-like clusters (Figure 2) and was designated HCT C, while the other grew as tightly packed, polygonal shaped cells (Figure 3) and was designated HCT C Col. Once the primary cultures on feeder layers almost totally covered the flasks, the process of weaning the malignant cells from the fibroblasts was commenced. As previously described, limited trypsinization was utilized to initiate cultures containing a mixture of fibroblasts and malignant cells (Brattain *et al.*, 1981*b*). After the secondary flasks grew to near confluency, cells forming grape-like clusters were easily removed from the cultures by mechanical manipulations and transferred to new flasks. The newly inoculated flasks contained a higher ratio of malignant cells to fibroblasts than the flasks from which they were transferred. This process was repeated several times until flasks of morphologically pure malignant cells growing in grape-like clusters were obtained. These cells were designated HCT C cells (Figure 2). The malignant cells growing in tight epithelial-like clusters were obtained by trypsinization of the flasks from which

**Figure 1.** Phase contrast microscopy of HCT C cells growing on human fibroblasts (M cells). (M $\times$ 100.)

**Figure. 2.** Phase contrast microscopy of HCT C cells growing in grape-like cluster. (M $\times$ 100.)

large numbers of the grape-like clusters had been removed. Fibroblasts were more easily removed by trypsinization than the malignant cells. Repeated removal of the fibroblasts by this procedure finally left cultures which were morphologically free of fibroblasts. These cells were designated HCT C Col (Figure 3).

**Figure. 3.** Phase contrast microscpopy of HCT C col cells growing in a tightly-packed epithelial-like colony. (M × 100.)

*Characterization of malignant cells from tissue culture*

HCT C cells from culture grew quite readily in semi-solid medium and on confluent mouse fibroblasts (Table I). Essentially the same extent of growth was obtained with HCT C Col cells (data not shown). Colony formation on confluent fibroblasts was approximately 1.5 × higher than that observed for 0.5% agarose after 1 week of incubation. We wondered whether this difference was reflective of the orgin of cultured HCT C cells on M cells. Therefore, further experiments were performed to determine the effects of fibroblasts on colony formation by HCT C from culture and directly from xenografts of HCT C cells grown in athymic nude mice. When cultured HCT C cells were grown in soft agarose systems (1% under layer −0.5% upper layer containing the cell inoculum) over feeder layers of confluent mouse fibroblasts there was slight, but consistent increase of approximately 25% in colony formation after 1 week of incubation relative to agarose cultures without feeder layers (Table I). After 2 weeks of incubation this increase was more pronounced (~50%).

Malignant cells from xenografts showed a more dramatic increase in colony formation when they were plated directly on to confluent monlayers of mouse fibroblasts rather than flasks without feeder layers (Table II). There were approximately 3.3 × as many colonies on flasks with feeder layers 4 days after seeding cells from the xenografts. At 7 days the number of colonies had increased in flasks with or without feeder layers but those with feeder layers had ~4.3 × the number of colonies as flasks without feeder layers. Again, similar results were obtained with HCT C Col.

*CEA*

The amount of CEA secreted by confluent cultures of HCT C and HCT C Col was compared. CEA production (72 h.) in confluent cultures of HCT C

**Table I** Colony formation by cultured HCT C cells on confluent fibroblasts and in semi-solid medium

| *Assay system* | *Experiment* | *Colony formation[b] after 1 week (%)* | *Colony formation[b] after 2 weeks (%)* |
|---|---|---|---|
| I. Cells plated on confluent fibro-blasts. | A | 10.4 | Too confluent for microscopic colony scoring |
| | B | 10.2 | |
| | C | 10.2 | |
| II. Cells plated in 0.5% agarose without feeder layers. | A | 7.2 | 8.8 |
| | B | 6.2 | 6.2 |
| | C | 6.7 | 8.0 |
| III. Cells plated in 0.5% agarose with feeder layers. | A | 8.8 | 12.4 |
| | B | 7.8 | 10.6 |
| | C | 8.4 | 12.4 |

[a]Each experiment reflects colony scoring on a total of 1.0 $cm^{-2}$ in 5 different counts for each of 3 different cultures.

[b]colony formation is expressed as $\frac{\text{\# colonies observed}}{\text{\# cells plated}} \times 100\%$

**Table II** Colony formation by cells obtained from HCT C xenografts plated with and without fibroblast feeder layers

| *Assay System* | *Experiment*[a] | *Colony formation*[b] *after 4 days* | *Colony formation*[b] *after 7 days* |
|---|---|---|---|
| I. Cells plated | A | 0.1 | 0.3 |
| without feeder | B | 0.3 | 0.3 |
| layers | C | 0.2 | 0.3 |
| II. Cells plated | A | 0.8 | 1.3 |
| with feeder | B | 0.8 | 1.5 |
| layers | C | 0.6 | 1.2 |

[a]Each experiment reflects colony scoring on a total of 1.0 $cm^{-2}$ in 5 different counts for each of 3 different cultures

[b]Colony formation is expressed as $\frac{\text{colonies observed}}{\text{\# cells plated}} \times 100\%$

amounted to 24ng $10^{-6}$ cells. Production for confluent cultures of HCT Col was 442ng $10^{-6}$ cells.

### *Iodinated cell surface components*

Typical electrophoretic profiles for HCT C and HCT C Col are shown in Figures 4 and 5, respectively. Qualitatively the profiles are quite similar except in the range of 116–200K where HCT C Col has relatively minor peaks between 92.5 and 116K. HCT C has 2 major peaks between 66 and 92.5K. While HCT C Col has the same 2 peaks, the material with the lower apparent

**Figure. 4.** SDS electrophoretic pattern of $^{125}$I-labeled plasma membrane components of HTC C.

**Figure. 5.** SDS electrophoretic pattern of $^{125}$I-labeled plasma membrane components of HCT C col.

molecular weight is relatively minor. Qualitatively the material between 21.5 and 66K is very similar for both sub-lines, but the HCT C peaks in this range constitute a relatively larger portion of the recovered material.

### *Karyology*

The chromosome distributions are shown in Figures. 6 and 7. In HCT C, the modal number was 46 but there were 12% of the cells with a chromosome number 47. Karyotyping of the cells with 46 chromosomes indicated a normal karyotype at the banding level achieved in this study. However, the cells with 47 chromosomes revealed an extra number 7 chromosome (Figure 8*a*, *b*).

HCT C Col cells presented a modal chromosome number of 47. Karyotyping cells from HCT C Col revealed 47, XY, +7, in 90% of the G-banded cells

**Figure. 6.** Chromosomal distribution of HCT C.

**Figure. 7.** Chromosomal distribution of HCT C col.

**Figure. 8.** (a) Karyology of HCT C-46 chromosomes; (b) Karyology of HCT C-47 chromosomes; (c) Karyology of HCT C col.

(Figure 8*c*). The remaining 10% of the cells also showed an additional chromosome 7 with random loss of other chromosomes.

### Discussion

Two sub-lines of human colonic carcinoma have been isolated from the same primary tumour and subsequently characterized. HCT C Col appears to be a pure sub-line on the basis of its karyology. Approximately 90% of the cells contain 47 chromosomes (+7) and the 10% which do not have 47 chromosomes show a random loss with +7. The karyology of HCT C is considerably more heterogeneous than that of HCT C Col and suggests that ~10% of cells in HCT C may be HCT C Col due to the presence of an extra chromosome 7. In spite of their relatively small difference in karyology, the 2 sub-lines show extensive biological and biochemical differences in morphology, CEA production, and cell surface components. The relationship between the karyologies of the sub-lines and their other different properties is, of course, unknown.

Recently, however, Chen *et al*, (1982) reported karyotypic analysis of 9 human colorectal carcinoma cell lines carried in tissue culture. Chromosome 7 was over-represented in 8/9 lines and, in addition, this chromosome 7 was the site of the highest incidence of structural modifications observed in the lines. Genes for the receptor for epidermal growth factor and the histones have been assigned to chromosome 7. The biological and biochemical differences between the sub-lines we have observed may be related to the additional chromosome 7 found in HCT C Col and/or differences in the expression of other areas of the genome.

Interestingly, the sub-lines with the more aberrant karyology appeared to be the better differentiated of the two by morphology (Figure. 1). HCT C Col grew in distinct epithelial-like colonies while HCT C grew as loosely adherent, rounded cells. These patterns of growth were retained for both cell lines regardless of whether they were grown directly on plastic or on either type of feeder layer utilized in this study. This observation indicates that the morphologies of the 2 sub-lines were not due to the surfaces on which they were grown. The greater production of CEA by HCT C Col is also of interest in this regard since some investigations have suggested that CEA production may be linked to differentiation (Dexter & Hager, 1980).

Additional molecular differences between the two sub-lines were observed in the SDS-electrophoretic profiles of $^{125}$I-labelled surface components. Specific surface components have not been isolated and characterized, thus the diferrences may be of a more quantitative nature. Furthermore, the observed differences in cell surface components may be a reflection of differential accessibility to the radioiodination procedure rather than the presence of qualitatively different components. However, the observation of differences between the electrophoretic profiles of the plasma membrane components of the sub-lines raises the possibility that their different biological properties may be related to cell surface alterations.

Heterogeneity of solid tumours is an important phenomenon which has been addressed in recent reviews (Calabresi *et al.*, 1979; Poste & Fidler, 1980). The phenomenon has been described in human colonic carcinoma (Brattain *et al.*, 1977*a, b*; 1981*a, b*; Dexter *et al.*, 1979). Although C and C Col cells were recognized in primary cultures of a tumour specimen, they may have arisen as a result of selection pressures from *in vitro* culture. However, the resolution and culture of sub-lines from individual tumours provides a potential model system by which appropriate biochemical and/or immunological reagents could be developed for the purpose of identifying heterogeneous populations in primary tumours. It is important to note that since HCT C Col and HCT C were obtained from the same primary specimen this model system has the advantage that observed molecular differences should be related to the expression of histocompatibility or blood group antigens, a factor which might affect the comparison of cell lines derived from different individuals.

Supported by Grants CA 21520, CA 29495, CA 13148 from the National Cancer Institute and American Cancer Society Grant PDT-109B.

### References

BRATTAIN, M.G. (1979). Tissue disaggregation. In: Flow Cytometry and Sorting (M. Melamed, P. Mullaney, M. Mendelsohn, eds.) John Wiley, New York.

BRATTAIN, M.G., BRATTAIN, D.E., FINE, W.D., KHALED, F.M., MARKS, M.E., ARCOLANO, L.A. & DANBURY, B.H. (1981*a*) Initiation of cultures of human colonic carcinoma with different biological characteristics utilizing feeder layers of confluent fibroblasts. *Oncodev. Biol. and Med.*, **2**, 355–366.

BRATTAIN, M.G., FINE, W.D., KHALED, F.M., THOMPSON, J. & BRATTAIN, D.E. (1981*b*) Heterogeneity of malignant cells from a human colonic carcinoma. *Cancer Res.*, **41**, 1751–1756.

BRATTAIN, M.G., GREEN, C., KIMBALL, P.M., MARKS, M.E. & KHALED, F.M. (1979) Isoenzymes of $\beta$-hexosaminidase from normal rat colon and colonic carcinoma. *Cancer Res.*, **39**, 4083–4090.

BRATTAIN, M.G., KIMBALL, P.M., PRETLOW, T.G. & PITTS, A.M. (1977*a*) Partial purification of human colonic carcinoma cells by sedimentation. *Br. J. Can.*, **35**, 850–857.

BRATTAIN, M.G., PRETLOW, T.P. & PRETLOW, T.G. (1977*b*) Cell fractionation of large bowel cancer. *Cancer*, **40**, 2479–2486.

BRATTAIN, M.G., STROBEL-STEVENS, J., FINE, W.D., WEBB, M. & SARRIF, A.M. (1980) Establishment of mouse colonic carcinoma cell lines with different metastatic properties. *Cancer Res.*, **40**, 2142–2146.

CALABRESI, P., DEXTER, D.L. & HEPPNER, G.H. (1979) Clinical and pharmacological implications of cancer cell differentiation and heterogeneity. *Biochem. Pharmacol.*, **28**, 1933–1941.

CHEN, T.R., HAY, R.J. & MACY, M.L. (1982) Karyotype consistency in human colorectal carcinoma cell lines established *in vitro*. *Cancer Gen. and Cyto.*, **6**, 93–117.

DEXTER, D.L., BARBOSA, J.A. & CALABRESI, P. (1979) N,N-Dimethyl-formamide-induced alteration of cell culture characteristics and loss of tumorigenicity in cultures of human colon carcinoma cells. *Cancer Res.*, **39**, 1020.

DEXTER, D.L. & HAGER, J.C. (1980) Maturation-induction of tumour cells using a human colon carcinoma model. *Cancer*, **45**, 1178.

EPSTEIN, A.L. & KAPLAN, H.S. (1979) Feeder layer and nutritional requirements for the establishment and cloning of human malignant lymphoma cell lines. *Cancer Res.*, **39**, 1748.

FIDLER, I.J. (1978) Tumor heterogeneity and the biology of cancer invasion and metastasis. *Cancer Res.*, **38**, 2651.

GALLIE, B.L., HOLMES, W. & PHILLIPS, R.A. (1982) Reproducible growth in tissue culture of retinoblastoma tumor specimens. *Cancer Res.*, **42**, 301.

HEPPNER, G.H., DEXTER, D.L., DeNUCCI, T., MILLER, F.R. & CALABRESI, P. (1978) Heterogeneity in drug sensitivity among tumor cell subpopulations of a single mammary tumor. *Cancer Res.*, **38**, 3758.

KIMBALL, P.M. & BRATTAIN, M.G. (1978) A comparison of methods for the isolation of carcinoembryonic antigen. *Cancer Res.*, **38**, 619.

McKUSICK, V. (1980) The anatomy of the human genome. *J. Hered.*, **71**, 370.

POSTE, G. & FIDLER, I.J. (1980) The pathogenesis of cancer metastasis. *Nature*, **283**, 139.

SEABRIGHT, M. (1972) The use of proteolytic enzymes for the mapping of structural rearrangements in the chromosomes of man. *Chromosome, (Berl.)*, **36**, 204.

SMITH, H.S., LAN, S., CERIANI, R., HACKETT, A.J. & STAMPFER, M.R. (1981) Clonal proliferation of cultured nonmalignant and malignant human breast epithelia. *Cancer Res.*, **41**, 4637.

YUNIS, J.J. & CHANDLER, M. (1978) High resolution chromosome analysis in clinical medicine. *Prog Clin Pathol.*, **7**, 267.